# Photoluminescence Spectra Correlations with Structural Distortion in Eu^3+^- and Ce^3+^-Doped Y_3_Al_5-2*x*_(Mg,Ge)*_x_*O_12_ (*x* = 0, 1, 2) Garnet Phosphors

**DOI:** 10.3390/ma17102445

**Published:** 2024-05-19

**Authors:** Heonji Ha, Sungjun Yang, Sangmoon Park

**Affiliations:** 1 Department of Engineering in Energy Materials, Graduate School, Silla University, Busan 46958, Republic of Korea; 2 UNIST Central Research Facilities, Ulsan National Institute of Science and Technology, Ulsan 44919, Republic of Korea; 3 Department of Environmental Energy & Chemistry, College of Engineering and Department of Fire Protection and Safety Management, College of Health and Welfare, Silla University, Busan 46958, Republic of Korea

**Keywords:** garnet, X-ray diffraction, phosphor, distortion

## Abstract

Garnet-type materials consisting of Y_3_Al_5-2*x*_(Mg,Ge)*_x_*O_12_ (*x* = 0, 1, 2), combined with Eu^3+^ or Ce^3+^ activator ions, were prepared by a solid-state method to determine the structural and optical correlations. The structure of Y_3_Al_5-2*x*_(Mg,Ge)*_x_*O_12_ (*x* = 1, 2) was determined to be a cubic unit cell (Ia-3d), which contains an 8-coordinated Y^3+^ site with octahedral (Mg,Al)O_6_ and tetrahedral (Al,Ge)O_4_ polyhedra, using synchrotron powder X-ray diffraction. When Eu^3+^ or Ce^3+^ ions were substituted for the Y^3+^ site in the Y_3_Al_5-2*x*_(Mg,Ge)*_x_*O_12_ host lattices, the emission spectra showed a decrease in the magnetic dipole *f*-*f* Eu^3+^ transition and a redshift of the *d*-*f* Ce^3+^ transition, related to centrosymmetry and crystal field splitting, respectively. These changes were monitored according to the increase in Mg^2+^ and Ge^4+^ contents. The dodecahedral and octahedral edge sharing was identified as a key distortion factor for the structure-correlated luminescence in the Eu^3+^/Ce^3+^-doped Y_3_Al_5-2*x*_(Mg,Ge)*_x_*O_12_ garnet phosphors.

## 1. Introduction

Ce^3+^-doped Y_3_Al_5_O_12_ (YAG) phosphor has been widely utilized as a smart light source in conjunction with blue LED chips [1,2,3]. Initially developed in 1967 by G. Blasse and A. Bril, the yellow Ce^3+^-activated YAG phosphor was prepared for use in flying-spot cathode-ray tubes for color television, emitting intense yellow light via the 5*d*-4*f* transition of Ce^3+^ ions within the cubic garnet YAG structure [4]. The garnet mineral belongs to the nesosilicate subclass, characterized by isolated tetragonal polyhedra [5]. The YAG garnet structure, a cubic crystal system (Ia-3d), consists of dodecahedral YO_8_, octahedral AlO_6_, and tetrahedral AlO_4_ units. The local dodecahedral YO_8_ polyhedra within the garnet structure exhibit edge-sharing with YO_8_ and AlO_6_ polyhedra, as well as vertex-sharing with isolated AlO_4_ tetrahedra [6,7,8,9]. In garnet host lattices, Ce^3+^ activator ions can occupy the dodecahedral site, influencing crystal field splitting and resulting in a shift of the *d*–*f* transition [10,11,12]. Researchers J. Ueda and S. Tanabe have explored the effects of crystal and electronic structures on Ce^3+^-doped YAG, particularly regarding the crystal field splitting of the lowest 5*d* levels and a new distortion parameter estimated by the ratio of dodecahedral edges [6]. Similarly, Eu^3+^ activator ions provide insight into site-resolved luminescence, distinguishing between centrosymmetric and non-centrosymmetric sites in garnet-type phosphors using the magnetic dipole and electric dipole transitions observed in emission spectra [13,14,15]. In this study, the structure of single-phase Y_3_Al_5-2*x*_(Mg,Ge)*_x_*O_12_ (*x* = 1, 2) garnet materials was determined using synchrotron X-ray analysis, revealing a cubic unit cell with Ia$\bar{3}$d symmetry. The cell parameters, volume, and distances within the host lattices were discussed. By substituting Ce^3+^ or Eu^3+^ activator ions into Y_3_Al_5-2*x*_(Mg,Ge)*_x_*O_12_ (*x* = 0, 1, 2), correlations between emission spectra, dipole transitions, and crystal field splitting were investigated in relation to structural distortion parameters.

## 2. Materials and Methods

Garnet materials doped with Eu^3+^- or Ce^3+^-doped Y_3_Al_5-2*x*_(Mg,Ge)*_x_*O_12_ (*x* = 0, 1, 2) compounds were synthesized by mixing appropriate stoichiometric amounts of powdered Y_2_O_3_ (Alfa, 99.9%), MgO (Alfa, 99.95%), Al_2_O_3_ (Alfa, 99.95%), GeO_2_ (Alfa, 99.999%), Eu_2_O_3_ (Alfa, 99.9%), and CeO_2_ (Aldrich, 99.9%), along with 5 wt% LiF (Alfa, 99.98%) or Li_2_CO_3_ (Alfa, 99%) flux. The powdered precursors were mixed in an agate mortar and pestle and then heated at 950 °C and 1400 °C for 6 h in air in a box furnace. The obtained powder samples containing the CeO_2_ precursor underwent additional reheating at 1000 °C for 12 h in a 5% H_2_/95% N_2_ atmosphere. Phase identification was conducted using a powder diffractometer (Cu Kα radiation; Shimadzu XRD-6000, Kyoto, Japan), and structural analysis of the obtained garnet materials was performed using synchrotron powder X-ray diffraction (λ = 0.65303 Å). The new structural data were collected at the PLS-II 6D UNIST-PAL beamline of the Pohang Accelerator Laboratory (PAL) [16]. The garnet Y_3_Al_5-2*x*_(Mg,Ge)*_x_*O_12_ (*x* = 1 and 2) structures were refined using the Rietveld refinement program FullProf Suite [17,18]. Excitation and emission photoluminescence spectra of the powdered phosphors were obtained via UV spectroscopy using spectrofluorometers (FluoroMate FS-2, Scinco, Seoul, Korea).

## 3. Results and Discussion

**Figure 1:**
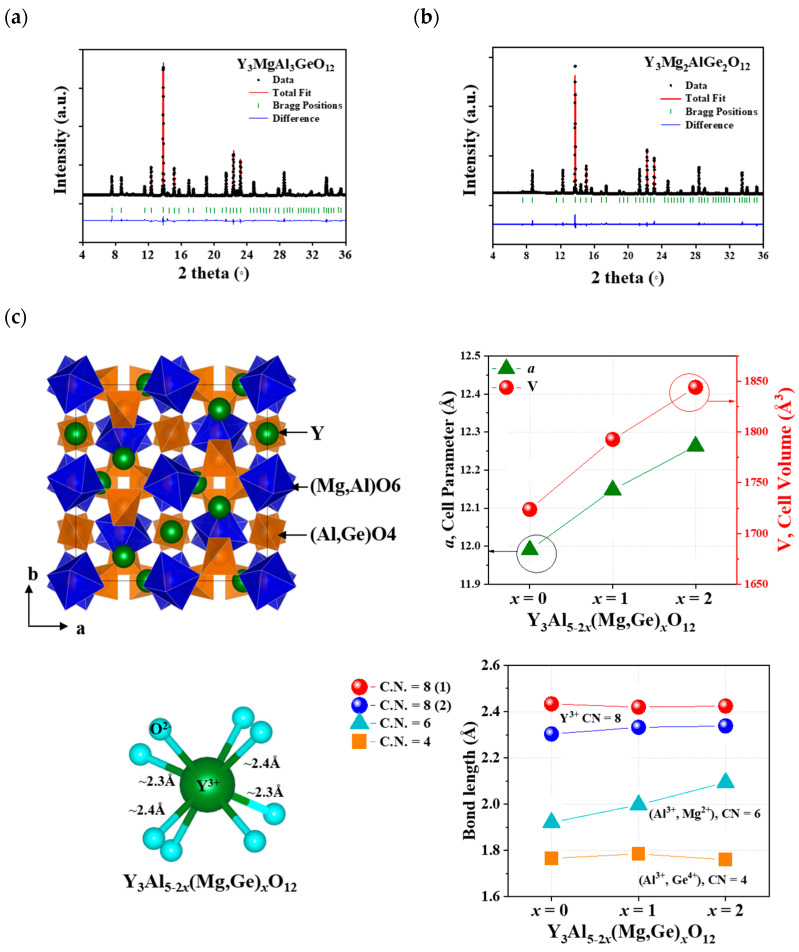
Synchrotron XRD patterns of Y_3_Al_5-2*x*_(Mg,Ge)*_x_*O_12_ (**a**) *x* = 1 and (**b**) *x* = 2 and (**c**) the garnet Y_3_Al_5-2*x*_(Mg,Ge)*_x_*O_12_ (*x* = 0, 1, 2) structure with cell parameters and cell volumes.

Figure 1a,b illustrate the Rietveld refinement fitting of the powdered X-ray diffraction (XRD) data for Y_3_Al_5-2*x*_(Mg,Ge)*_x_*O_12_ (*x* = 1 and 2). The summarized structural data are provided in Table 1, Table 2 and Table 3. A single phase of the garnet structure, determined to be a cubic unit cell (Ia$\bar{3}$d), was obtained through a solid-state reaction method. This cubic phase of garnet, including Y_3_Al_5_O_12_ (YAG, ICSD 170157) and Y_3_Al_5-2*x*_(Mg,Ge)*_x_*O_12_ (*x* = 1 and 2) structures, comprises 8-, 6-, and 4-coordinated Y^3+^, Al^3+^(1)-Mg^2+^, and Al^3+^(2)-Ge^4+^ ions, respectively, occupying 24c, 16a, and 24d Wyckoff sites. The 6-coordinated Mg^2+^ (with a radius of 0.72 Å for 6 coordination number (CN)), Al^3+^(1) (with a radius of 0.535 Å for 6 CN), and 4-coordinated Al^3+^ (with a radius of 0.39 Å for 4 CN) and Ge^4+^ (with a radius of 0.39 Å for 4 CN) sites are suitable for substitutions in the garnet structure [19]. Therefore, the formula for the garnet-structured Y_3_Al_5-2*x*_(Mg,Ge)*_x_*O_12_ (*x* = 0, 1, 2) can be expressed as Y_3_Al(1)_2-*x*_Mg*_x_*Al(2)_3-*x*_Ge*_x_*O_12_ based on the ionic radii of the cations in the unit cell, as depicted in Figure 1c. Similar to the isolated AlO_4_ tetrahedra in the YAG structure, the tetrahedral (Al,Ge)O_4_ polyhedron in the Y_3_Al_5-2*x*_(Mg,Ge)*_x_*O_12_ structures is also isolated, with no sharing of O atoms. There are two different bond distances between Y^3+^ and O^2−^ ions in the YO_8_ polyhedron, whereas a single bond distance is observed in the (Mg,Al)O_6_ and (Al,Ge)O_4_ polyhedra within the host lattices. The YO_8_ polyhedron shares edges with nearby YO_8_ polyhedra and (Mg,Al)O_6_ octahedra. YAG exhibits lattice parameters and volume, such as *a* = 11.9900(14) Å and V = 1723.68 Å^3^ (as shown in Table 1). The cell parameters and volumes of Y_3_Al_5-2*x*_(Mg,Ge)*_x_*O_12_ (*x* = 1 and 2) are larger than those of YAG compounds, such as Y_3_MgAl_3_GeO_12_ (*a* = 12.1479(2) Å and V = 1796.82(17) Å^3^) and Y_3_Mg_2_AlGe_2_O_12_ (*a* = 12.2628(1) Å and V = 1844.027(14) Å^3^). The Y–O bond distances of an 8-coordinated Y (with a radius of 1.019 Å) comprise four long distances (2.433 Å) and four short distances (2.303 Å) in the YAG structure. The Y–O bond distances in Y_3_MgAl_3_GeO_12_ and Y_3_Mg_2_AlGe_2_O_12_ host lattices remain consistent with those of the YAG structure, as shown in Figure 1c. However, the bond distances of a 6-coordinated (Mg,Al)-O_6_ and Mg-O_6_ exhibit distinct increases from 1.921 Å (for Al-O in YAG) to 1.997 Å and 2.094 Å, respectively, representing 4% and 9% differences. Conversely, the bond distances of Al–O_4_ and (Al,Ge)-O_4_ tetrahedra in the garnet structures remain similar, ranging from 1.766 to 1.785 Å and 1.760 Å. Interestingly, with the increase in the cell parameter and volume, the bond distance increase is observed primarily in the octahedral polyhedron in the Y_3_Al_5-2*x*_(Mg,Ge)*_x_*O_12_ structure when *x* = 0, 1, and 2.

**Figure 2:**
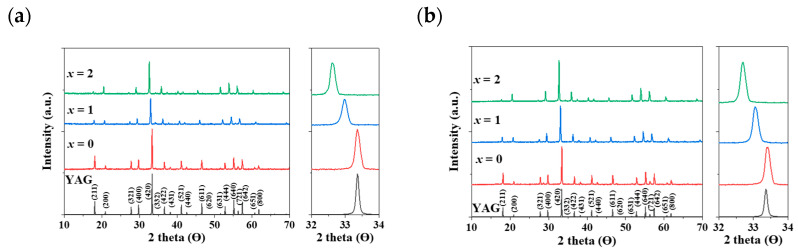
The calculated XRD pattern of YAG with Miller indices and obtained XRD patterns: (**a**) Eu^3+^-doped and (**b**) Ce^3+^-doped Y_3_Al_5-2*x*_(Mg,Ge)*_x_*O_12_ (*x* = 0, 1, 2) phosphors.

**Figure 3:**
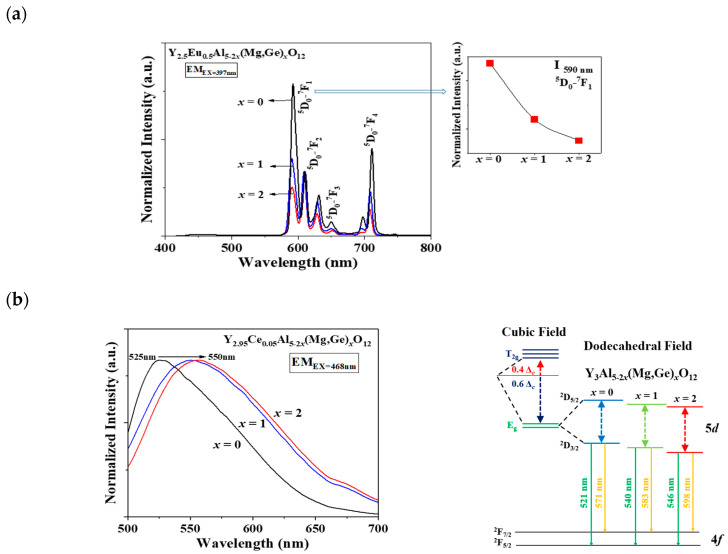
The emission spectra of (**a**) Eu^3+^-doped and (**b**) Ce^3+^-doped Y_3_Al_5-2*x*_(Mg,Ge)*_x_*O_12_ (*x* = 0, 1, 2) phosphors with relative centrosymmetric intensity and energy-level diagram.

In Figure 2a,b, a distinct shift in the apparent peaks to lower angles, particularly those at 2θ = 32–34°, was observed as Al^3+^ ions were gradually replaced by Mg^2+^ and Ge^4+^ ions in the Eu^3+^- and Ce^3+^-doped Y_3_Al_5-2*x*_(Mg,Ge)*_x_*O_12_ (*x* = 0, 1, 2) phosphors, respectively. A single phase of the garnet structure with a cubic crystal system (Ia$\bar{3}$d) was obtained, free from any apparent impurities. The cell volumes of both Y_2.5_Eu_0.5_Al_5-2*x*_(Mg,Ge)*_x_*O_12_ and Y_2.95_Ce_0.05_Al_5-2*x*_(Mg,Ge)*_x_*O_12_ (*x* = 0, 1, 2) phosphors increased as the content of Mg^2+^ and Ge^4+^ increased. Figure 3a displays the emission photoluminescence (PL) spectra of Y_2.5_Eu_0.5_Al_5-2*x*_(Mg,Ge)*_x_*O_12_ (*x* = 0, 1, 2) phosphors. The electronic *f*-*f* transitions of Eu^3+^ ions in the host lattices are assigned as ^5^D_0_–^7^F_1_, ^5^D_0_–^7^F_2_, ^5^D_0_–^7^F_3_, and ^5^D_0_–^7^F_4_ within the range of 550 and 750 nm [13,14,15]. It is known that when Eu^3+^ ions are located at the centrosymmetric site in a crystal structure, the magnetic dipole transition (^5^D_0_–^7^F_1_) dominates, whereas, in the absence of inversion Eu^3+^ ions in the host lattice, the electric dipole transition (^5^D_0_–^7^F_2_) dominates [13,14,15]. The centrosymmetric symmetry of the local-environment-center Eu^3+^ ions in the Y_3_Al_5-2*x*_(Mg,Ge)*_x_*O_12_ structure was inferred from the normalized intensity ratio of the magnetic dipole transition, as depicted in Figure 3a, Figures S1 and S2. All intensity values in the emission spectra were normalized by dividing them by the maximum intensity value of the electric dipole transition peak. As Eu^3+^ ions occupy the 8-coordinated Y^3+^ site in the Y_3_Al_5-2*x*_(Mg,Ge)*_x_*O_12_ (*x* = 0, 1, 2) structures, the dominant magnetic dipole transition around 590 nm was noticeably decreased up to *x* = 0, 1, and 2. This indicates that the ideal cubic field, characterized by a centrosymmetric Y^3+^ center (D_4h_ point group), was gradually distorted to a dodecahedral field with the substitution of Mg^2+^ and Ge^4+^ ions. Figure 3b presents the emission spectra of Y_2.95_Ce_0.05_Al_5-2*x*_(Mg,Ge)*_x_*O_12_ (*x* = 0, 1, 2) phosphors (S1 and 2) and relative energy diagrams of cubic and distorted cubic polyhedrons, showcasing the 8-coordinated site geometry for the 5*d* Ce^3+^ orbital energy levels in the host materials. Upon substituting Mg^2+^ and Ge^4+^ ions for Al^3+^ ions in the structure, the distorted 8-coordinated Ce^3+^ ions in the YAG exhibit a significant redshift of the e_g_ orbital splitting from a normal cubic polyhedron, resulting in emissions from the red-shifted 5*d* energy level caused by the high-crystal-field effect [10,11,12]. 

**Figure 4:**
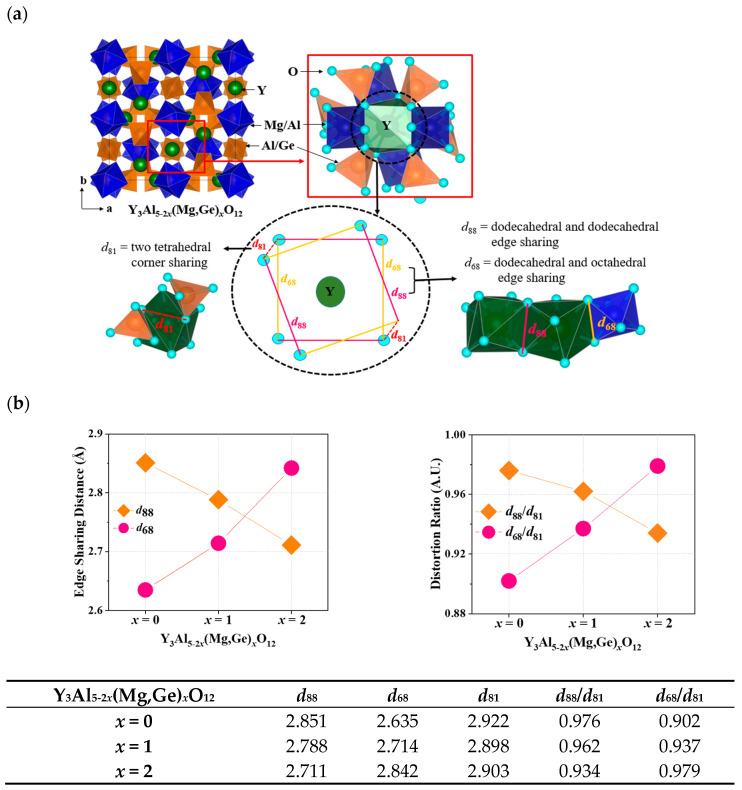
(**a**) Unit cell and local structure of the dodecahedral site in Y_3_Al_5-2*x*_(Mg,Ge)*_x_*O_12_ (**b**) distortion parameters of *d*_88_, *d*_68_, *d*_81_, *d*_88_/*d*_81_, and *d*_68_/*d*_81_ for Ce^3+^-doped Y_3_Al_5-2*x*_(Mg,Ge)*_x_*O_12_ (*x* = 0, 1, 2) phosphors.

In Figure 4a, the unit cell and local structure of the dodecahedral site in Y_3_Al_5-2*x*_(Mg,Ge)*_x_*O_12_ are depicted. Ce^3+^ ions engaged in the Y^3+^ site of garnet structures can emit light due to the distortion factor expressed by the ratio of dodecahedral edges. The four short and long Y(Ce)-O bond distances in the garnet structures can be influenced by the deviation of O-O bonds shared with adjacent dodecahedra (*d*_88_) and two non-shared tetrahedra (*d*_81_) [6]. This deviation in the local structural arrangement around the Ce^3+^ ions within the garnet lattice can have an impact on the energy levels of the Ce^3+^ ions [6]. As distortion increases due to the compression on the cube, the maximum emission shifts to longer wavelengths according to the lower excited state of energy level in the cubic crystal field splitting. Figure 4b summarizes distortion parameters including *d*_88_, *d*_68_, *d*_81_, *d*_88_/*d*_81_, and *d*_68_/*d*_81_ for Ce^3+^-doped Y_3_Al_5-2*x*_(Mg,Ge)*_x_*O_12_ (*x* = 0, 1, 2) phosphors. The edge-sharing distance of *d*_88_ and distortion parameter of *d*_88_/*d*_81_ gradually decreased by substituting Mg-Ge ions in the structures, indicating that the compression of the cube was released by the decrease in distortion. However, the maximum emission wavelength of the Ce^3+^ *d*-*f* transition shifts to longer wavelengths, as shown in Figure 3. Furthermore, the centrosymmetric Eu^3+^ emission decreased when *x* = 0 to 1 and 2 in Eu^3+^-doped Y_3_Al_5-2*x*_(Mg,Ge)*_x_*O_12_ garnet phosphors. When Mg^2+^-Ge^4+^ ions were doped into the Y_3_Al_5_O_12_ structure, the bond length of Mg-O among Y-O, Al-O, and Ge-O bonds from the structure analysis solely increased with increasing cell volumes, as shown in Figure 1c. For the distorted Eu^3+^ and Ce^3+^ cubic sites in the Y_3_Al_5-2*x*_(Mg,Ge)*_x_*O_12_ garnet structures, the parameter of dodecahedral and octahedral edge-sharing should be estimated as a distortion factor. Figure 4b illustrates the steady increase in edge-sharing distance *d*_68_ and distortion ratio of *d*_68_/*d*_81_ in terms of key distortion parameters, resulting in the non-centrosymmetric manners and the redshift of the maximum emission wavelength.

## 4. Conclusions

Eu^3+^/Ce^3+^-doped Y_3_Al_5-2*x*_(Mg,Ge)*_x_*O_12_ (*x* = 0, 1, 2) phosphors were synthesized using a solid-state reaction method assisted by excess LiF or Li_2_CO_3_ flux at high temperature. Synchrotron powder X-ray diffraction analysis confirmed that the single-phase of Y_3_Al_5-2*x*_(Mg,Ge)*_x_*O_12_ (*x* = 1 and 2) garnet materials possessed a cubic unit cell (Ia$\bar{3}$d) with lattice parameters of *a* = 12.1479(2) Å and *a* = 12.2628(1) Å, respectively. These garnet structures consisted of an 8-coordination environment for Y^3+^ with (Mg^2+^,Al^3+^(1))O_6_ octahedra and (Al^3+^(2),Ge^4+^)O_4_ tetrahedra. Interestingly, the distance value of the Mg,Al(1)O_6_ octahedra only increased with the doping of Mg^2+^ and Ge^4+^ ions into the YAG host lattice. Upon doping Eu^3+^ or Ce^3+^ ions into the Y_3_Al_5-2*x*_(Mg,Ge)*_x_*O_12_ (*x* = 0, 1, 2) garnet structure, the resulting phosphors exhibited increases in non-centrosymmetric and strong crystal field splitting manners with increasing contents of Mg^2+^ and Ge^4+^ ions. This indicated that the cubic polyhedra were compressed by the increase in distortion. The distortion factor of dodecahedral–dodecahedral edge sharing/non-edge sharing (*d*_88_/*d*_81_) decreased with the substitution of Mg^2+^-Ge^4+^ ions, while the distortion factor of dodecahedral–octahedral edge sharing/non-edge sharing (*d*_68_/*d*_81_) gradually increased. In Y_3_Al_5-2*x*_(Mg,Ge)*_x_*O_12_ (*x* = 0, 1, 2) garnet structures, when substituting ions in octahedral and tetrahedral sites, the dodecahedral–octahedral edge sharing/non-edge sharing (*d*_68_/*d*_81_) should be considered as the most crucial distortion parameter.

## Figures and Tables

**Table 1:** Rietveld refinement and crystal data for Y_3_Al_5-2*x*_(Mg,Ge)*_x_*O_12_ (*x* = 0, 1, 2).

Chemical Formula	Y_3_Al_5_O_12_ (ICSD 170157)	Y_3_MgAl_3_GeO_12_	Y_3_Mg_2_AlGe_2_O_12_
Radiation type, λ (Å)		Synchrotron (6D-BM), 0.65303	
2θ range (deg)		4–36	
Crystal system	Cubic	Cubic	
Space group	Ia-3d	Ia-3d	
Lattice parameter (Å)	*a* = 11.9900 (14)	*a* = 12.1479 (2)	*a* = 12.2628 (1)
Volume (Å^3^)	V = 1723.68	V = 1792.682 (17)	V = 1844.027 (14)
Density (g/cm^3^)	4.57	4.888	4.816
R*_p_* (%)		0.717	0.728
R*_wp_* (%)		1.22	1.47
R*_exp_* (%)		1.26	1.88
*S*		0.96	0.78
χ^2^		0.931	0.608

**Table 2:** Refined atomic coordinates of Y_3_Al_5-2*x*_(Mg,Ge)*_x_*O_12_ (*x* = 0, 1, 2).

**Y_3_Al_5_O_12_ (ICSD 170157)**						
**Atom**	**Wyckoff position**	**x**	**y**	**z**	**B_iso_**	**SOF**
Y	24*c*	0.125	0	0.25	0.00365 (12)	1
Al(1)	16*a*	0	0	0	0.0030 (3)	1
Al(2)	24*d*	0.375	0	0.25	0.0011 (3)	1
O	96*h*	0.28023 (17)	0.10110 (16)	0.19922 (17)	0.0036 (4)	1
**Y_3_MgAl_3_GeO_12_**						
**Atom**	**Wyckoff position**	**x**	**y**	**z**	**B_iso_**	**SOF**
Y	24*c*	0	0.25	0.125	0.33 (8)	1
Mg	16*a*	0	0	0	0.49	0.5
Al(1)	16*a*	0	0	0	0.49	0.5
Al(2)	24*d*	0	0.25	0.375	0.57	0.6667
Ge	24*d*	0	0.25	0.375	0.57	0.3333
O	96*h*	−0.0305 (5)	0.0551 (6)	0.1518 (6)	0.4 (2)	1
**Y_3_Mg_2_AlGe_2_O_12_**						
**Atom**	**Wyckoff position**	**x**	**y**	**z**	**B_iso_**	**SOF**
Y	24*c*	0	0.25	0.125	0.2 (2)	1
Mg	16*a*	0	0	0	1.0 (2)	1
Al(2)	24*d*	0	0.25	0.375	0.1 (2)	0.3333
Ge	24*d*	0	0.25	0.375	0.1 (2)	0.6667
O	96*h*	−0.0315 (7)	0.0576 (6)	0.1576 (6)	0.2 (2)	1

**Table 3:** Selected interatomic distances for Y_3_Al_5-2*x*_(Mg,Ge)*_x_*O_12_ (*x* = 0, 1, 2).

Y_3_Al_5_O_12_ (ICSD 170157)		Y_3_MgAl_3_GeO_12_		Y_3_Mg_2_AlGe_2_O_12_	
Atom	Distance (Å)	Atom	Distance (Å)	Atom	Distance (Å)
Y-O (×4)	2.433	Y-O (×4)	2.419 (2)	Y-O (×4)	2.424 (4)
Y-O (×4)	2.303	Y-O (×4)	2.332 (2)	Y-O (×4)	2.338 (4)
Al-O (×6)	1.921	(Mg/Al1)-O (×6)	1.997 (2)	Mg-O (×6)	2.094 (4)
Al-O (×4)	1.766	(Al2/Ge)-O (×4)	1.785 (2)	Ge/Al-O (×4)	1.760 (4)

## Data Availability

The authors state that data supporting the study in the manuscript are available on reasonable request. Informed consent was obtained from all subjects involved in the study.

## Supplementary Materials

The following supporting information can be downloaded at: https://www.mdpi.com/article/10.3390/ma17102445/s1, Figure S1: The X-ray diffraction patterns and excitation and emission spectra of Eu^3+^- and Ce^3+^-doped Y_3_Al_5-2x_(Mg,Ge)_x_O_12_ ((A) x = 0, (B) x =1, (C) x =2) phosphors.; Figure S2: The excitation and emission spectra of (A) Y_2.5_Eu_0.5_Mg_x_Al_5-2x_Ge_x_O_12_ and (B) Y_2.95_Ce_0.05_Mg_x_Al_5-2x_Ge_x_O12 (x = 0–2).
