# Supplementary material for: Photoluminescence Spectra Correlations with Structural Distortion in Eu3+- and Ce3+-Doped Y3Al5-2x(Mg,Ge)xO12 (x = 0, 1, 2) Garnet Phosphors
Source: Materials (Basel). 2024 May 19;17(10):2445. doi: 10.3390/ma17102445 (PMC11123408; doi:10.3390/ma17102445)

Supporting Information

Photoluminescence spectra correlations with structural distortion in Eu^{3+}- and Ce^{3+}-doped $Y_3Al_{5-2x}(Mg,Ge)_xO_{12}$ (x = 0, 1, 2) garnet phosphors

Heonji Ha[1], Sungjun Yang[2], and Sangmoon Park[1,3,*]

[1]Department of Engineering in Energy Materials, Graduate School, Silla University, Busan 46958, Republic of Korea

[2]UNIST Central Research Facilities, Ulsan National Institute of Science and Technology, Ulsan 44919, Republic of Korea

[3]Department of Environmental Energy & Chemistry, College of Engineering and Department of Fire Protection and Safety Management, College of Health and Welfare, Silla University, Busan 46958, Republic of Korea

***Corresponding author:**

E-mail: spark@silla.ac.kr

Figure S1. The X-ray diffraction patterns and excitation and emission spectra of Eu^{3+}- and Ce^{3+}-doped $Y_3Al_{5-2x}(Mg,Ge)_xO_{12}$ ((A) $x = 0$, (B) $x = 1$, (C) $x = 2$) phosphors.

(A)

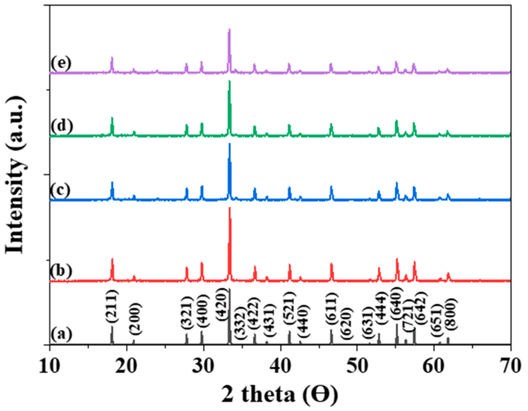

The calculated XRD patterns of $Y_3Al_5O_{12}$ (a) and obtained XRD patterns of $Y_{3-p}Eu_pAl_5O_{12}$ phosphors (b): p = 0.1, (c) p = 0.3, (d) p = 0.5, and (e) p = 0.7.

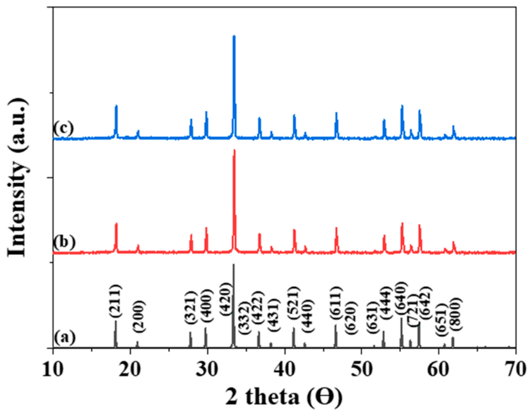

The calculated XRD patterns of $Y_3Al_5O_{12}$ (a) and obtained XRD patterns of $Y_{3-q}Ce_qAl_5O_{12}$ phosphors: (b) q = 0.01 and (c) q = 0.05

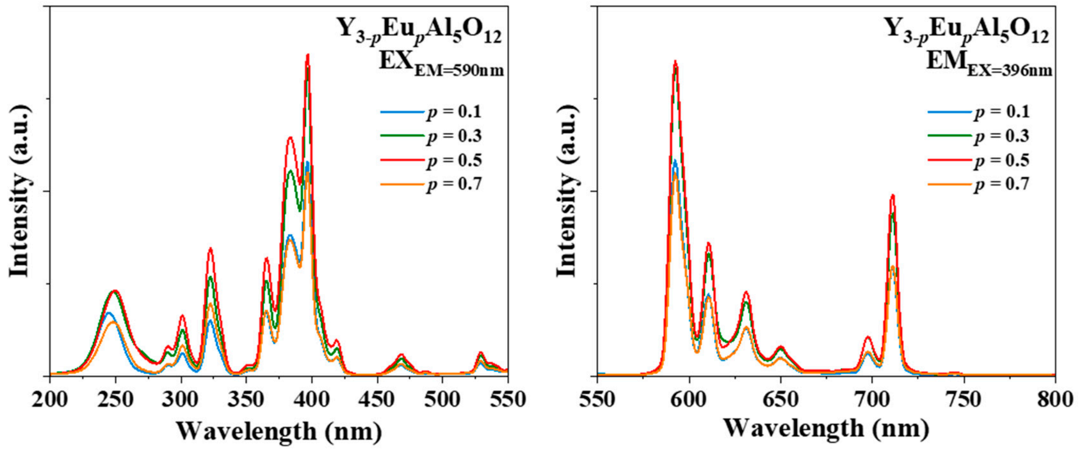

The excitation and emission spectra of $Y_{3-p}Eu_pAl_5O_{12}$ (p = 0.1-0.7).

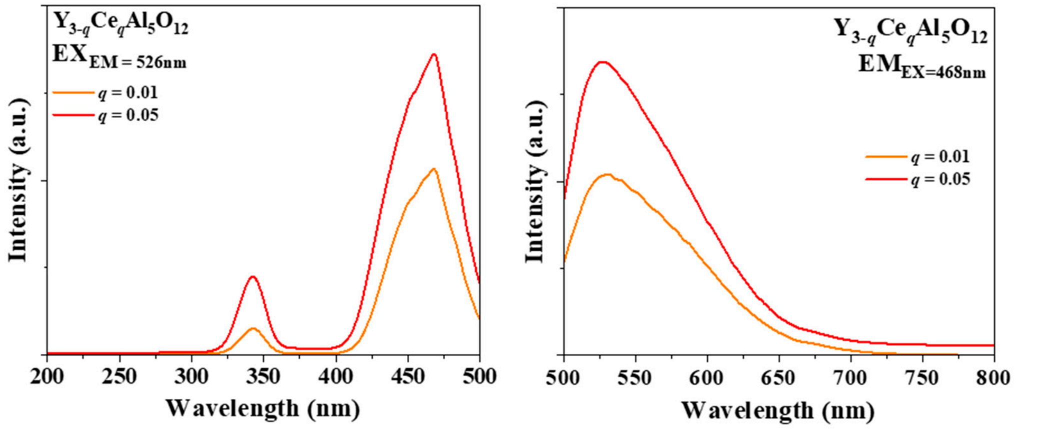

The excitation and emission spectra of $Y_{3-q}Ce_qAl_5O_{12}$ (q = 0.001-0.05).

(B)

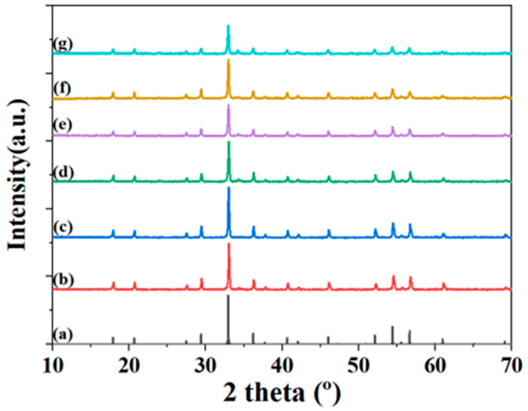

The calculated XRD patterns of $Y_3MgAl_3GeO_{12}$ (a) and obtained XRD patterns of $Y_{3-p}Eu_pMgAl_3GeO_{12}$ phosphors (b): p = 0.01, (c) p = 0.1, (d) p = 0.3, (e) p = 0.5, (f) p = 0.7, and (d) p = 0.8.

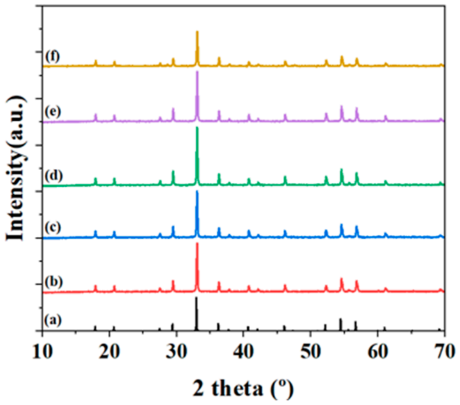

The calculated XRD patterns of $Y_3MgAl_3GeO_{12}$ (a) and obtained XRD patterns of $Y_{3-q}Ce_qMgAl_3GeO_{12}$ phosphors (b): q = 0.001, (c) q = 0.005, (d) q = 0.01, (e) q = 0.03, and (f) q = 0.05.

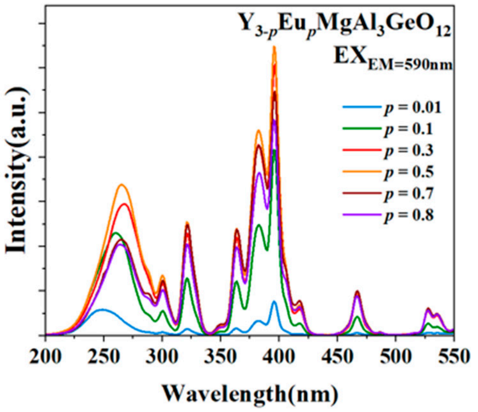

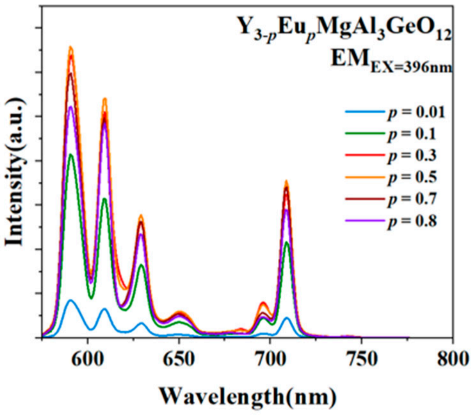

The excitation and emission spectra of $Y_{3-p}Eu_pMgAl_3GeO_{12}$ (p = 0.01-0.8).

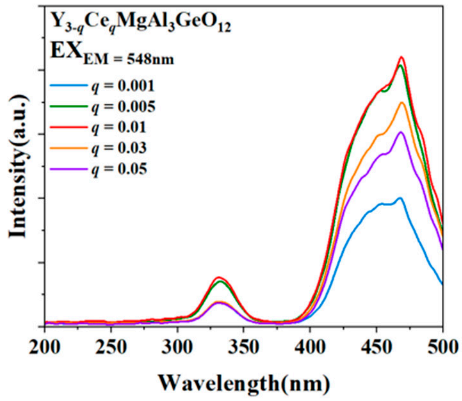

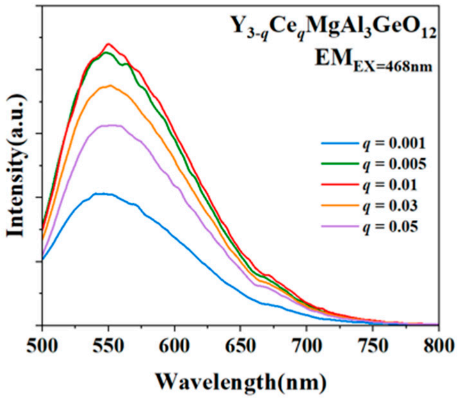

The excitation and emission spectra of $Y_{3-q}Ce_qMgAl_3GeO_{12}$ (q = 0.001-0.05).

(C)

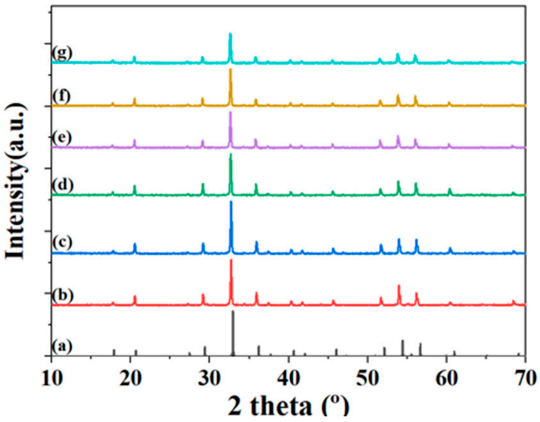

The calculated XRD patterns of $Y_3Mg_2AlGe_2O_{12}$ (a) and obtained XRD patterns of $Y_{3-p}Eu_pMg_2AlGe_2O_{12}$ phosphors (b): $p = 0.01$, (c) $p = 0.1$, (d) $p = 0.3$, (e) $p = 0.5$, (f) $p = 0.6$, and (d) $p = 0.8$.

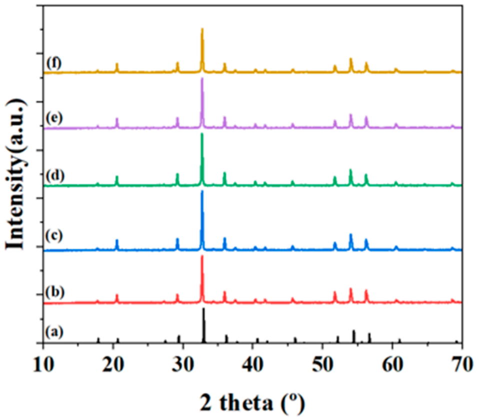

The calculated XRD patterns of $Y_3Mg_2AlGe_2O_{12}$ (a) and obtained XRD patterns of $Y_{3-q}Ce_qMg_2AlGe_2O_{12}$ phosphors (b): $q = 0.001$, (c) $q = 0.005$, (d) $q = 0.01$, (e) $q = 0.025$, and (f) $q = 0.05$.

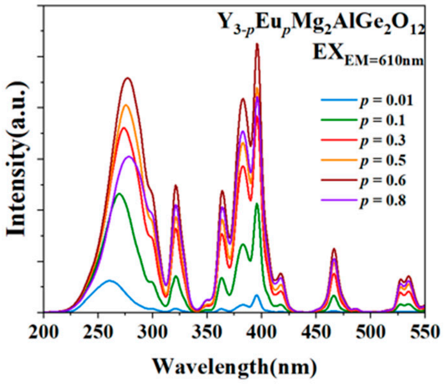

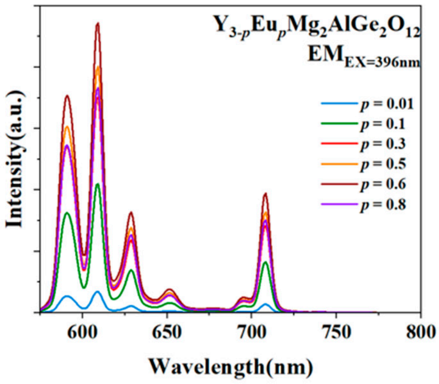

The excitation and emission spectra of $Y_{3-p}Eu_pMg_2AlGe_2O_{12}$ ($p = 0.01$-0.8).

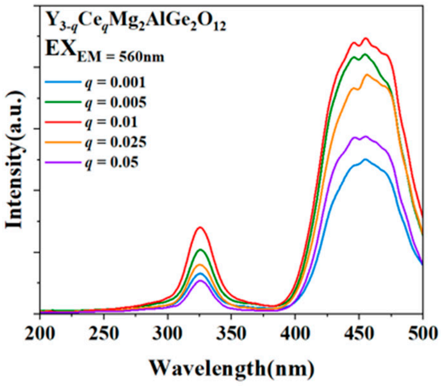

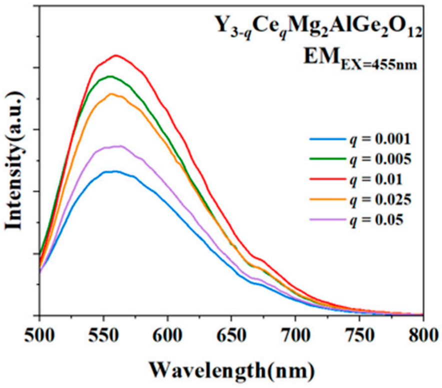

The excitation and emission spectra of $Y_{3-q}Ce_qMg_2AlGe_2O_{12}$ ($q = 0.001$-0.05).

Figure S2. The excitation and emission spectra of (A) $Y_{2.5}Eu_{0.5}Mg_xAl_{5-2x}Ge_xO_{12}$ and (B) $Y_{2.95}Ce_{0.05}Mg_xAl_{5-2x}Ge_xO_{12}$ (x = 0-2).

(A)

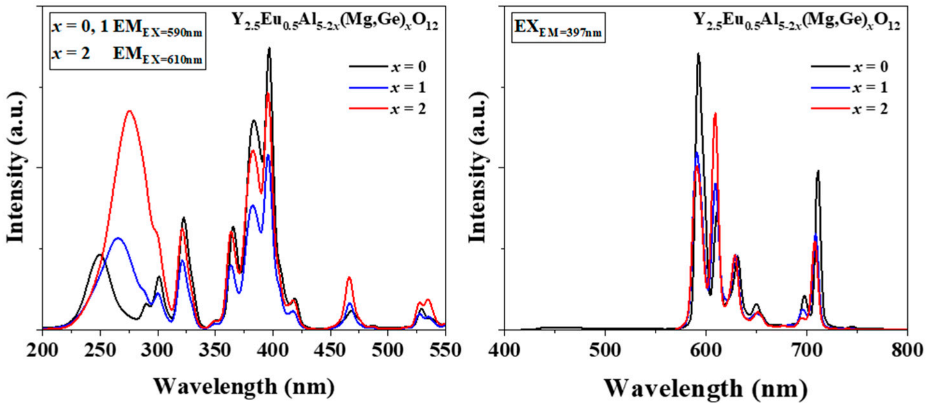

(B)

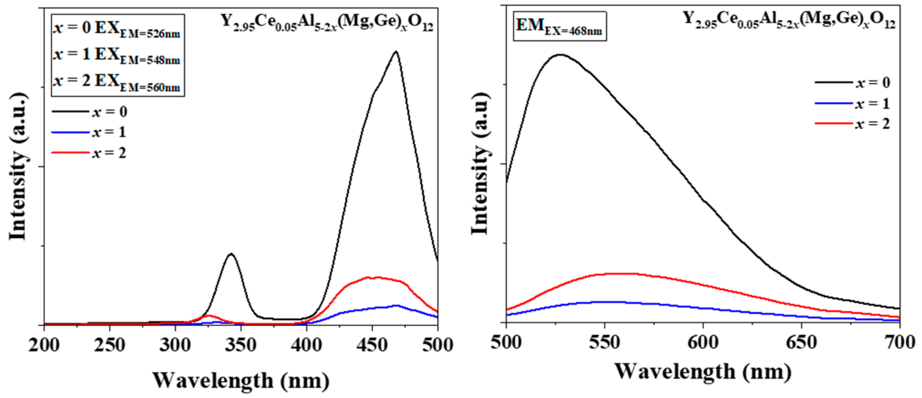

Supplement: Supplementary file 1 [file materials-17-02445-s001.zip › materials-3003739-supplementary.pdf]
